# Application and development of stimulus-responsive hydrogel biomaterials for diabetic wound healing: a literature review

**DOI:** 10.3389/fmed.2025.1740559

**Published:** 2026-01-08

**Authors:** Xin Jin, Weiqiang Lan, Wei Yong, Zhifa Yuan, Xiaojun Li, Xixi Cui

**Affiliations:** 1 Department of Orthopedics, Dazhou Dachuan District People’s Hospital (Dazhou Third People’s Hospital), Dazhou, Sichuan, China; 2 Department of Orthopedics, Nanchong Traditional Chinese Medicine Hospital, Nanchong, Sichuan, China; 3 Department of Orthopedics, Beijing Anzhen Nanchong Hospital of Capital Medical University & Nanchong Central Hospital, Nanchong, Sichuan, China; 4 Department of Endocrinology, Dazhou Central Hospital, Dazhou, Sichuan, China

**Keywords:** diabetic wounds, chronic wound healing, hydrogel, stimulus response, wound

## Abstract

Diabetic wounds (DWs) are a severe complication of diabetes, defined as the presence of ulcers or deep tissue damage below the ankle in diabetic patients. DWs entail enormous psychological and economic burdens on diabetic patients. The microenvironment of diabetic wound healing is highly complex, characterized by hyperglycemia, excessive inflammation and elevated reactive oxygen species (ROS), making wound repair and healing extremely challenging. Traditional wound dressings have single function and insufficient adaptability to the wound environment, making it difficult to meet the complex needs of the healing process. As an emerging biomaterial, hydrogels offer distinct advantages over traditional dressings in terms of water absorption, breathability, biocompatibility, drug-carrying capacity, and environmental responsiveness, making them a subject of significant interest in the repair of diabetic wounds. Based on the diabetic wound microenvironment, we summarize and discuss hydrogel wound dressings responsive to temperature, pH, glucose, reactive oxygen species, enzymes, and multiple stimuli, integrating recent research advances in stimulus-responsive wound dressings.

## 1 Introduction

Diabetes mellitus (DM) is a chronic disease characterized by elevated blood glucose levels, leading to metabolic disorders with significant consequences (1, 2). The number of adults with diabetes worldwide has exceeded 800 million, having increased more than fourfold since 1990 and is expected to increase to 1.31 billion by 2050 (3, 4). Among the various complications associated with DM, diabetic foot ulcers (DFUs) represent a major clinical challenge due to their complexity and poor healing (5). DFU is one of the serious complications leading to disability and death in diabetic patients, which is highly prevalent, difficult and costly to treat, resulting in a serious health and economic burden worldwide (6). Alarmingly, approximately 25% of DFU require amputation within 6 to 18 months of diagnosis, which is 10 to 20 times higher than in nondiabetic individuals (7–10). The healing process of DWs is often impeded, or even completely stalled, due to factors such as excessive inflammation, oxidative stress, peripheral neuropathy, and impaired angiogenesis in the local wound environment (11, 12). In the clinical management of these wounds, dressings play a pivotal role. However, traditional dressings often possess singular biochemical functions and are incapable of precise active substance delivery, which limits their efficacy in the treatment of DWs. In contrast, stimuli-responsive multifunctional hydrogel dressings, which can achieve controlled release of active substances in response to changes in the wound microenvironment, hold significant potential for the therapeutic management of these wounds (13).

Recently, several comprehensive reviews have summarized the synthesis and general applications of stimuli-responsive hydrogels (14–17). For instance, Khattak et al. and Hu et al. provided excellent overviews of endogenous and exogenous stimuli-responsive mechanisms, while Xu et al. focused on fabrication methodologies. Chen et al. highlighted potential clinical properties. Although numerous stimuli-responsive hydrogels have shown promise in preclinical models, a significant gap remains between laboratory research and clinical translation. Current reviews often focus heavily on material synthesis or single-stimulus mechanisms, neglecting the critical analysis of why these advanced materials struggle to replace traditional dressings in standard clinical practice. Furthermore, the “fundamental mismatch” between the static nature of conventional treatments and the highly dynamic, complex microenvironment of DWs (characterized by fluctuating glucose, pH, and ROS levels) requires a more systematic discussion. This review addresses these gaps by not only summarizing multi-stimuli-responsive strategies but also critically evaluating the barriers to clinical adoption, providing a bridge between material science innovation and clinical reality.

## 2 Factors impeding diabetic wound healing

The physiological process of wound healing consists of four sequential yet overlapping phases: hemostasis, inflammation, proliferation, and remodeling. The many cells and components involved in these processes work together to rebuild damaged skin tissue and restore its integrity (18). The hemostatic and inflammatory phases are mainly associated with wound healing and pathogen resistance, respectively, which is achieved through the production and action of platelets, neutrophils and macrophages (19). During the proliferative phase, secreted factors increase vascular permeability and edema, and endothelial, epidermal and dermal cells accumulate around the wound site due to the release of growth factors and cytokines (20). The proliferative phase progresses to angiogenesis, when fibroblasts and collagen synthesis increase, culminating in the formation of granulation tissue (21). After 1–3 weeks, tissue remodeling occurs through the differentiation of fibroblasts into myofibroblasts, an increase in type I collagen, and extracellular matrix (ECM) recovery (22, 23). The newly formed vascular network rapidly develops into a mature tissue structure with limited structural strength and a reduced number of resident cells, ultimately leading to scar tissue formation (24).

Although the wound healing process also consists of these four stages in diseases such as diabetes, a complex microenvironment can impede progress and lead to chronic wound formation (25, 26).

## 3 Pathophysiological mechanisms and microenvironments of diabetic wound

On one hand, in diabetic patients, persistent hyperglycemia leads to the development of distal lower extremity neuropathy, encompassing motor, autonomic, and sensory neuropathy. Motor neuropathy can result in biomechanical abnormalities and foot deformities. Autonomic neuropathy can cause alterations in skin viscoelasticity, such as dryness and cracking. Sensory neuropathy, however, can lead to a loss of protective sensation (6). This reduction or absence of sensation renders the nervous system insensitive or even unaware of injuries, significantly increasing the risk of ulceration and even amputation. Another primary mechanism of DWs is angiopathy, which commonly affects arteries below the knee. This vascular disease can lead to stenosis or even complete occlusion of the arterial lumen, causing local ischemia, insufficient blood perfusion, and hypoxia in the lower extremities. This, in turn, can trigger lower limb ulceration and gangrene. Furthermore, these vascular lesions may also cause or exacerbate the patient’s loss of sensation (27). During weight-bearing, trauma or excessive long-term localized pressure on the foot can lead to the formation of DFUs. Subsequently, bacterial infections and the formation of biofilms further complicate the wound microenvironment, causing the ulcer to persist and resist healing (28).

**Figure 1:**
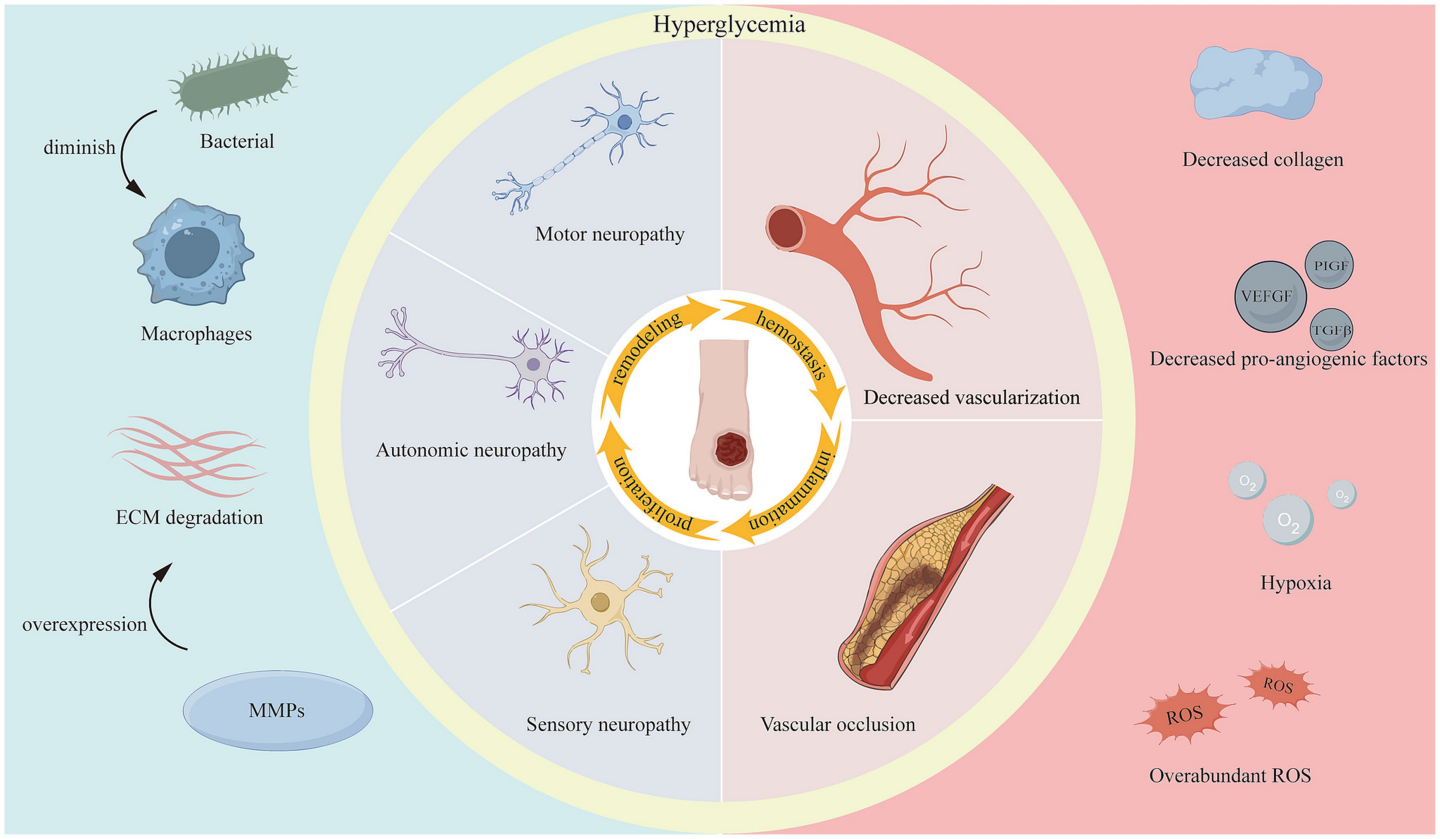
Factors impeding diabetic wound healing.

On the other hand, the DWs microenvironment is quite complex and is characterized by hyperglycemia, hypoxia, excessive wound exudate, recurrent bacterial infections, ROS accumulation, disturbed cytokine and growth factor expression, increased protease activity, persistent inflammation, and impaired angiogenesis and tissue regeneration (29–32). All of these factors impede the healing process, prolong one or more of the four overlapping phases of wound healing (33) (Figure 1), and increase the difficulty of DWs treatment.

### 3.1 High glucose level

Clinically, hyperglycemia at the site of injury not only leads to cellular damage and vasculopathy, but also significantly increases the risk of bacterial infection. For example, formulations of advanced glycosylation end products (AGEs) can directly activate immune cells to produce high levels of ROS, ultimately leading to elevated oxidative stress, disruption of cellular redox balance and increased metabolic disturbances in the wound area (34–36). In addition, AGEs can disrupt macrophage conversion from typically activated (M1) macrophages with pro-inflammatory properties to alternatively activated (M2) macrophages with anti-inflammatory and tissue repair functions (37). Excessive M1 macrophage infiltration leads to persistent aggregation and activation of pro-inflammatory cells at the site of the lesion, resulting in prolonged duration of inflammation (38). Elevated blood glucose levels also impair healing by hardening cell membranes and vasoconstriction, which reduces blood flow and deprives the wound site of nutrients and oxygen (39).

In addition to this, elevated blood glucose levels can provide additional nutritional resources for bacterial growth and proliferation, which can lead to recurrent bacterial infections (40). High blood glucose levels are one of the most prominent features of the DWs environment and therefore the significant barrier to healing. Therefore, the primary requirement in the clinical management of DWs is the control of blood glucose levels. Schneider et al. (41) summarized several clinical studies showing that chronic and infected wounds remain in an alkaline pH environment. However, the majority of pH-responsive hydrogel wound dressings reported to date have been designed to disintegrate at a low pH, possibly due to the consideration that bacteria at diabetic wound sites can convert glucose to lactic acid, thus creating a lower pH environment (29). Furthermore, this contradiction may be due to differences between animal models and diabetic patients. When the environmental pH is acidic, DWs in experimental animals are shorter in duration and may remain in the acute phase. This situation is different from the environment of clinical diabetic patients in the chronic phase (pH > 7.3) (41). Finally, the pH of a wound depends on the time course and the stage of the wound, e.g., chronic wounds can also exhibit an acidic pH during the healing process.

Collectively, the pH of diabetic wound sites varies widely and is related to many factors such as microbial colonization and the stage of wound healing progression. Therefore, designing wound dressings that precisely regulate the pH of DWs can significantly facilitate the wound healing process.

### 3.2 Wide and varying pH

The wound pH is dynamic and is affected by a variety of variables during the wound healing process that can influence bacterial infection, enzyme activity, oxygenation, cell proliferation, and other factors. To resist microbial invasion, the pH of the skin surface is usually weakly acidic at 4–6 (41). When the skin is damaged, the subcutaneous tissue is exposed and the internal pH environment is weakly alkaline at 7.4. This usually changes from alkaline to acidic as the wound heals, but the pH of chronic and infected wounds remains alkaline for a longer period of time due to ongoing inflammation.

Dissemond et al. (42) evaluated the pH of 39 patients with chronic wounds from various causes and found pH values ranging from 5.45 to 8.65. As with acute wounds, the pH of DWs usually starts as alkaline, progresses to neutral, and then changes to an acidic state (39), however, prolonged inflammation usually results in a decrease in the pH of the wound bed (43). The initial pH of the microenvironment is higher in chronic wounds compared to normal skin, which is more favorable for bacterial growth and colonization, increasing the risk of long-term bacterial infection (44).

### 3.3 Hypoxia

Clinical reports have shown that oxygen is critical in the wound healing process, which has become a therapeutic approach to aid and accelerate wound healing. The regulation of energy metabolism, oxidative stress and bacterial resistance are important at all stages of wound healing and are influenced by oxygen levels in the microenvironment (45). In DWs, capillary damage and impaired angiogenesis lead to inadequate oxygen supply and decreased immune response, which in turn exacerbates bacterial infection and local inflammation (46). In clinical settings, hyperbaric oxygen therapy is used to treat patients with diabetic foot when necessary to promote wound healing and reduce the risk of amputation in patients (47, 48). Notably, hyperbaric oxygen therapy remains an expensive treatment. Therefore, it may also be valuable to design convenient wound dressings that take full account of their effect on oxygen in the wound environment.

### 3.4 High levels of ROS

An excess of reactive oxygen species (ROS) is another critical factor contributing to impaired diabetic wound healing. Normal levels of ROS act as a second messenger for many immune cells and non-lymphocytes, effectively promoting angiogenesis and resistance to bacterial infection. However, prolonged excessive levels of ROS lead to chronic inflammation and irreversible cellular damage in the microenvironment, making wounds more fragile and inhibiting the function of endogenous stem cells and macrophages, impeding wound healing (49, 50). In DWs, oxidative stress-induced inflammation leads to collagen and ECM degradation and impaired angiogenesis (51). In addition to this, high levels of ROS produced by immune cells in wounds activate nuclear factor-κB (NF-κB) and significantly increase the expression of inflammatory mediators interleukin-6 (IL-6) and tumor necrosis factor-*α* (TNF-α), leading to chronic inflammation and slowing wound healing. Therefore, maintaining ROS at appropriate levels at the wound site may help promote wound healing.

### 3.5 Metalloproteinase overexpression

MMP has a dual role in diabetic wound healing. MMP is a gelatinase produced in the dermis after injury and plays an important role in ECM catabolism and tissue reorganization during the healing process (52). However, overexpression of MMP inhibits the production of early connective granulation tissue that fills the wound and inactivates growth factors that are essential for the wound healing process (30, 53). Overexpression of ECM proteases in wounds reduces the accumulation of ECM in diabetic wounds, which prevents wound closure and increases the risk of bacterial infection and chronic inflammation (54, 55). While scientists have demonstrated that overexpression of MMPs (e.g., MMP-2 and MMP-9) (56) impedes the wound healing process, other studies have shown that other MMPs (e.g., MMP-8) favor diabetic wound healing (57). Therefore, selective inhibition of matrix protease function is critical for diabetic wound healing. Notably, the levels of active MMP in DWs may differ significantly from those of diabetic mice used in the experiment.

Briefly, chronic DWs have a complex microenvironment (58), including excess exudate (59), hypoxia (60), bacterial infection (61), excess glucose (62), high levels of ROS (63), and overexpression of MMPs (64). All of these can impede the clinical healing of diabetic wounds and are challenges that must be overcome in clinical management.

## 4 Advantages of hydrogel dressings for the treatment of diabetic wound

DWs have a high incidence, and these long-term, non-healing wounds place a significant psychological and financial burden on patients. Conventional wound dressings commonly used in clinical practice, such as gauze and films, are unable to respond to changes in the wound environment, cannot provide sustained release of active substances, and do not accelerate the healing process. Furthermore, they carry the risk of adhering to the wound bed, which can cause secondary damage upon removal. Hydrogels are a class of hydrophilic, three-dimensional porous network systems composed of natural and/or synthetic polymers. Their structure is analogous to the ECM of many tissues, making them widely applicable in biomedical fields such as tissue engineering and drug delivery (65). Beyond their structural benefits, hydrogels function as versatile depots for a wide array of therapeutic agents, ranging from small-molecule drugs (e.g., antibiotics, anti-*inflammatory* agents) to macromolecules (e.g., growth factors like VEGF and EGF, nucleic acids) and even living cells. The high water content of hydrogels mimics the native tissue environment, effectively preserving the bioactivity of fragile proteins and preventing their denaturation (66). Therapeutic payloads can be incorporated into the *matrix* via various strategies, including physical encapsulation, electrostatic interaction, and covalent conjugation. While physical entrapment offers simplicity, it often suffers from an initial ‘burst release.’ In contrast, chemical immobilization allows for more sustained release kinetics governed by polymer degradation or stimuli-triggered bond cleavage, ensuring prolonged therapeutic concentrations that match the temporal requirements of the chronic wound healing process (67). However, conventional hydrogel wound dressings cannot achieve on-demand release of active substances, thus failing to meet the complex needs of DWs. The release process of active substances from stimuli-responsive hydrogel wound dressings can be modulated in response to changes in the external environment (e.g., UV light, magnetic fields, and ultrasound) or the local wound microenvironment (e.g., temperature, pH, glucose, enzymes, and ROS) (68, 69). This has established them as one of the most attractive and promising candidates for treating DWs. Therefore, stimuli-responsive hydrogel wound dressings hold immense application potential in the field of DWs repair.

## 5 Stimuli-responsive hydrogel dressings

**Figure 2:**
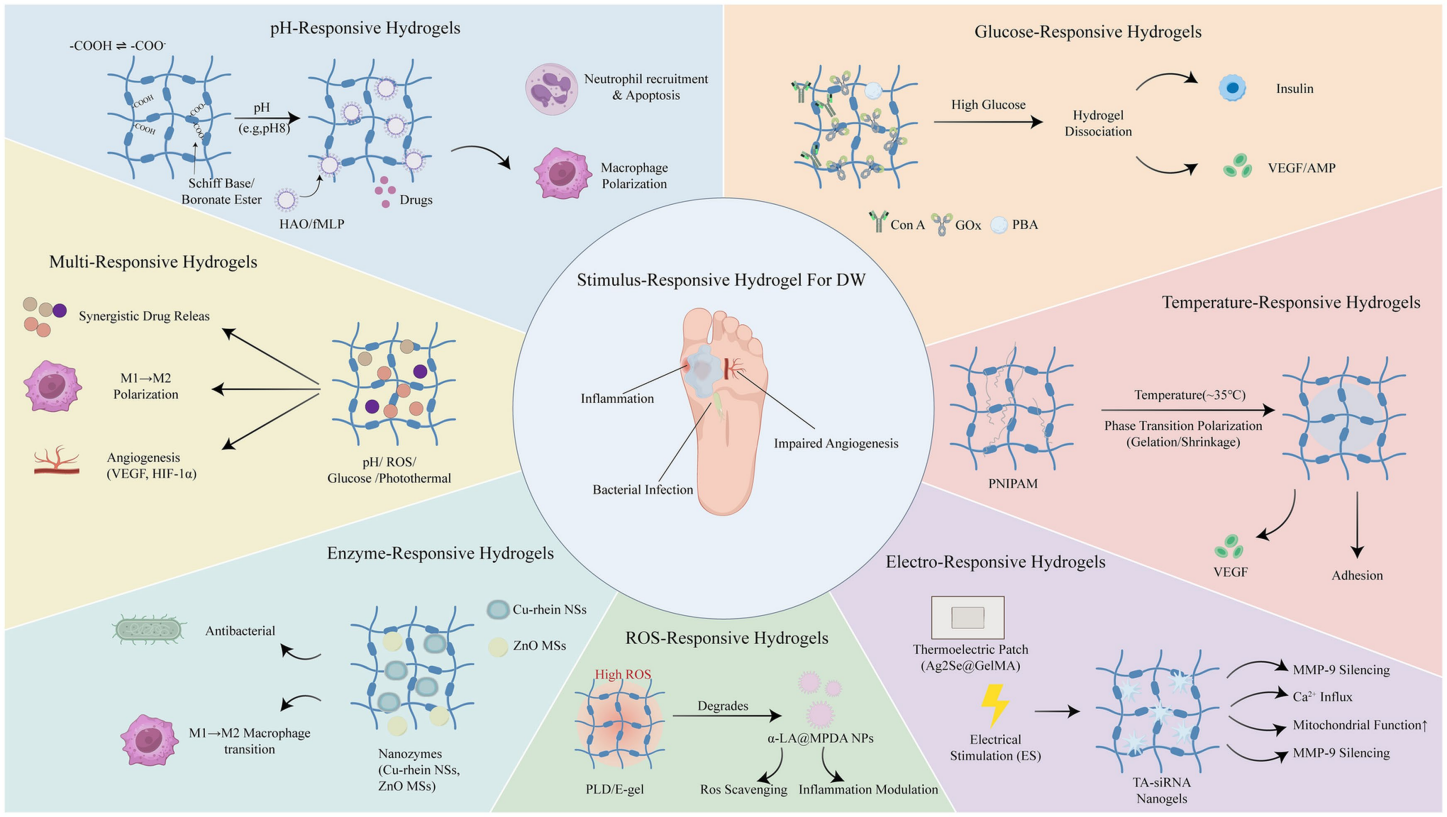
Overview of different stimulus-responsive hydrogel biomaterials applied in diabetic wound healing.

The complex pathological environment often involves distinct molecular pathways, rendering the microenvironment of DWs exceedingly intricate. Moreover, the cells and growth factors participating in the various stages of healing are subject to dynamic changes. Traditional hydrogel wound dressings, which release active substances passively, are ill-equipped to meet the complex demands of DWs applications. Therefore, in the construction of hydrogel dressings, structures responsive to changes in the external environment (e.g., ultraviolet, near-infrared light, magnetic fields, and ultrasound) or the wound microenvironment (e.g., pH, enzymes, ROS, and temperature) can be incorporated to regulate the release of active substances. Stimuli-responsive hydrogel wound dressings can precisely fulfill the requirements for antibacterial, anti-inflammatory, and pro-angiogenic actions at different stages of wound healing. In comparison to traditional passive dressings, stimuli-responsive hydrogels can enhance the healing rate of DWs, effectively lower the recurrence rate, reduce patient pain and economic burden, and thus demonstrate significant promise for clinical applications in wound care. To provide a comprehensive overview (Figure 2), Representative stimuli-responsive hydrogel systems are summarized in Table 1 (see Supplementary Table S1 for a comprehensive list with detailed mechanisms).

**Table 1:** Summary of representative stimuli-responsive hydrogel systems for diabetic wound healing.

Stimulus	Key material composition	Mechanism	Reference
Glucose	Concanavalin A (Con A)	Dissociation via competitive glucose binding to Con A.	Yin et al. (73)
	Glucose oxidase (GOx)	GOx-catalyzed acidification triggers Schiff base hydrolysis.	Wang et al. (75)
	Phenylboronic acid-modified gelatin (AP), Sodium alginate (SA)	Competitive binding to PBA groups disrupts boronate ester bonds.	Zhou et al. (77)
pH	Alginate calcium	Swelling via electrostatic repulsion of carboxyl groups at pH 8.0.	Wang et al. (83)
	Chitosan, 4-Formylphenylboronic acid (FPBA)	Acid-triggered hydrolysis of borate ester bonds via neutrophil recruitment.	Liu et al. (84)
	Incorporating carbon quantum dots (CQDs)	pH-dependent fluorescence/color changes for wound monitoring.	Yang et al. (86)
	Phenylboronic acid & benzaldehyde functionalized polymer (PEGS-PBA-BA)	Hydrolysis of Schiff base bonds at acidic pH (5.5).	Liang et al. (43)
	Oxidized Hyaluronic Acid (HA-ALD), Hydrazide Hyaluronic Acid (HA-HYD)	Hydrolysis of dynamic hydrazone bonds at wound pH (~6.5).	Jia et al. (87)
Temperature	N-isopropylacrylamide (NIPAM)	PNIPAM phase transition and shrinkage at 37 °C.	Chen et al. (92); Song et al. (93)
	Lauric acid (LA), stearic acid (SA)	LA/SA low eutectic mixtures dissolve above 39 °C.	Zhang et al. (96)
Electric	Polyvinyl alcohol (PVA), Human-like collagen (HLC), TA, Borax	Electric field drives release of charged nanogels.	Lei et al. (98)
	Ag₂Se, Gelatin Methacryloy (GelMA)	Thermoelectric potential generation via Ag₂Se and temperature difference.	Qin et al. (99)
ROS	Sulfhydryl-modified hyaluronic acid (HA-SH)	Oxidation and cleavage of disulfide bonds by ROS	Sun et al. (101)
	Ferrocene, β-Cyclodextrin (β-CD)	ROS oxidizes Ferrocene (Fe^2+^) to Ferrocenium (Fe^3+^), disrupting host-guest crosslinks with β-CD to trigger hydrogel dissociation and payload release	Tan et al., (102)
Enzyme	Oxidized Sodium Alginate (OSA), Borax, Gelatin.	Dissociation of Enzyme and Schiff base/borate ester bonds in acidic environment.	Feng et al. (106)
	HA grafted with the MMP-sensitive peptide sequence: CPLGLAG-NH-NH2	MMP-2 enzymatically cleaves the CPLGLAG peptide crosslinks.	Li et al., (62)
Multi	Oxidized dextran (OXD), Phenylboronic acid-modified carboxymethyl chitosan (CMCS-PBA), Polydopamine nanoparticles (PDANP)	Bond cleavage triggered by pH/Glucose/ROS; NIR-induced photothermal effect.	Dai et al. (109)
	NIPAM, 3-(acrylamido)phenylboronic acid (MPBA), Insulating elastomer (VHB)	Resistance changes triggered by temperature (NIPAM) and glucose (MPBA)	Guo et al. (110)

### 5.1 Glucose-responsive hydrogels

Persistent hyperglycemia in DWs is a primary driver of non-healing, as it promotes bacterial proliferation, induces the formation of AGEs, and impairs macrophage phagocytosis (70). Conventional dressings cannot actively regulate this parameter. To bridge this gap, glucose-responsive hydrogels are designed not merely as drug carriers, but as active metabolic modulators. By utilizing mechanisms such as Glucose Oxidase (GOx) catalysis or Phenylboronic Acid (PBA) competitive binding, these systems consume local glucose to break the vicious cycle of hyperglycemia while triggering the “on-demand” release of insulin or angiogenic agents.

**Figure 3:**
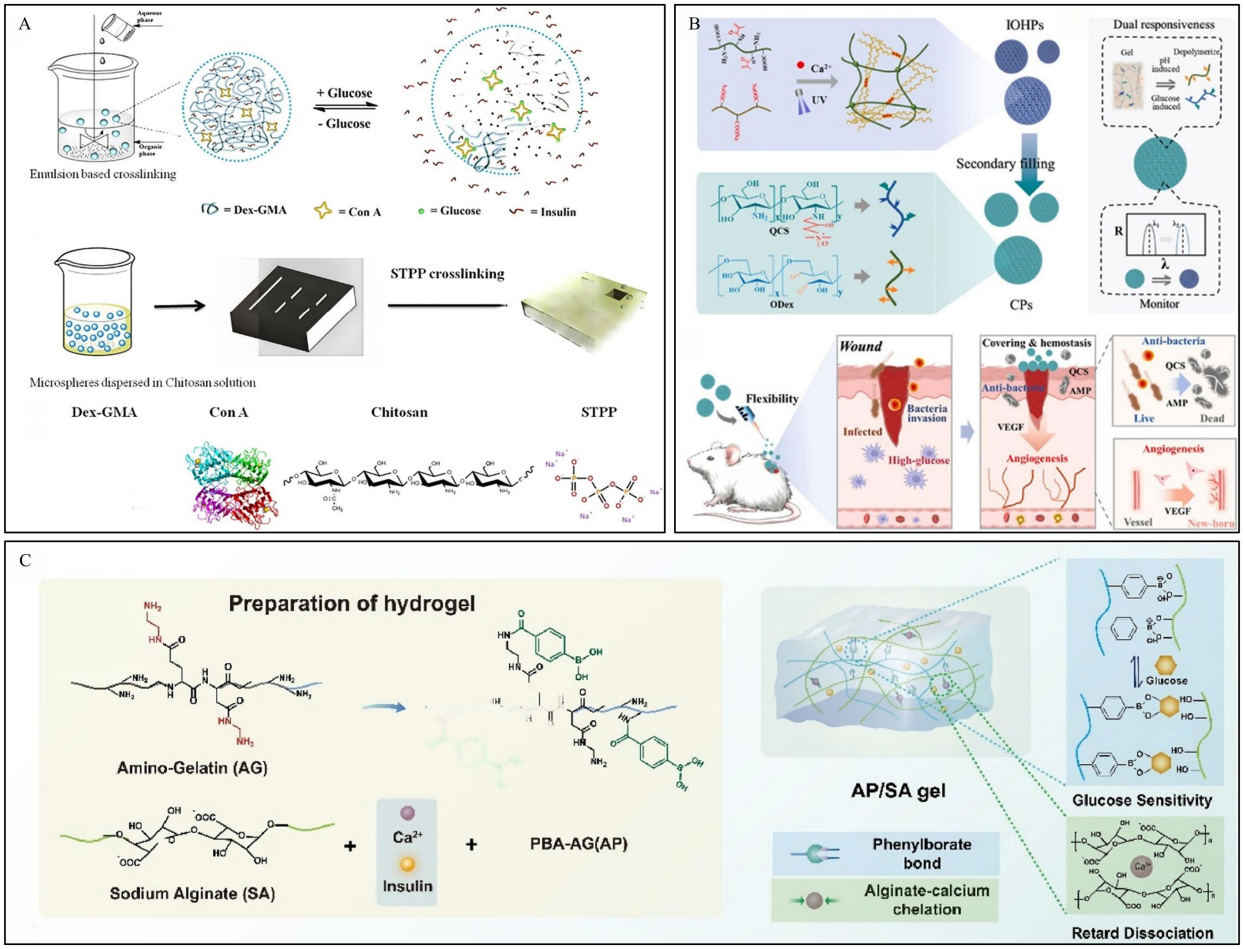
Construction process and structure of representative glucose-responsive hydrogels. **(A)** Fabrication process and glucose-responsive structural change of microspheres. Reproduced from ref. (73) Copyright 2019 Elsevier. **(B)** Schematic of CPs preparation and their application in DWs. Reproduced from ref. (75) Copyright 2025 Elsevier. **(C)** Fabrication process of AP/SA gel. Reproduced from ref. (77) Copyright 2026 Elsevier.

Con A, a protein-based plant lectin, is commonly used in glucose-responsive hydrogel systems. The Con A tetramer, composed of two dimers, possesses four binding sites for non-reducing polysaccharides (71). Free glucose can competitively bind with Con A within the hydrogel, leading to the dissociation of the hydrogel system, which forms the basis of this responsive mechanism (72). Yin et al. (73) developed hydrogel microspheres based on the crosslinking between methacrylated dextran, chitosan, and Con A, and loaded them with insulin (Figure 3A). Experiments demonstrated that the gel maintained a stable and sustained release of insulin even after 12 days, and the released insulin retained its activity, showing the potential of this hydrogel for application in chronic diabetic wound repair.

GOx can not only directly regulate insulin secretion, but also oxidize glucose in the wound area into gluconic acid, thereby lowering the microenvironment’s pH (74). Wang et al. (75) developed a hydrogel composed of an oxidized dextran/quaternized chitosan (ODex/QCS) hydrogel loaded with antimicrobial peptides (AMP), vascular endothelial growth factor (VEGF), and GOx, which was filled into a hyaluronic acid methacrylate/sodium alginate (HAMA/Alg) inverse opal skeleton. In the high-glucose environment of the wound, GOx catalyzes the oxidation of glucose to produce acidic products. This leads to a decrease in the local microenvironmental pH, promoting the degradation of the ODex/QCS hydrogel and the controlled release of AMP and VEGF (Figure 3B).

In the two aforementioned responsive systems, both GOx and Con A are protein-based substances. They are sensitive to the external environment, have a short storage life, and can easily elicit an immune response within the human body. In contrast, hydrogels based on PBA crosslinking exhibit low toxicity, good stability, and do not cause immune rejection, thus garnering extensive research and attention. PBA can reversibly bind with glucose and other compounds containing cis-diols to form boronate ester bonds (76). The high glucose concentration in DWs can competitively bind with PBA, leading to the dissociation of the hydrogel and promoting the release of drugs from the hydrogel system. Based on dynamic boronate ester bonds, Zhou et al. (77) developed a dual-crosslinked hydrogel, the AP/SA gel. Amino-functionalized gelatin grafted with phenylboronic acid (AP) and sodium alginate (SA) were crosslinked via dynamic boronate ester bonds, achieving high glucose concentration-responsive insulin release (Figure 3C). Furthermore, the introduction of calcium ions to chelate the carboxyl groups of SA significantly suppressed network hydrolysis, extending the drug delivery time to over 24 h while ensuring the intelligent delivery of insulin.

However, stability remains a critical challenge for clinical translation. These challenges stem from insufficient physiologic response of the dressing to adapt to the complex microenvironment of the wound. Glucose-responsive hydrogel dressings have the inherent ability to carry drugs and improve the wound microenvironment, promoting synergistic therapeutic outcomes. Nonetheless, targeted modifications are essential to create hydrogels that respond to glucose levels. For example, PBA-based hydrogels require improved glucose selectivity, while protein-based hydrogels (involving GOx and Con A) require enhanced protein stability (78–80)

### 5.2 pH -responsive hydrogels

Normal skin maintains an acidic mantle (pH 4–6) as a protective barrier (41). In contrast, chronic diabetic wound exudates often exhibit an alkaline pH (ranging from 7.4 to 8.9) due to persistent bacterial colonization and the metabolic activity of ammonia-producing pathogens. The pH of DWs usually starts as alkaline and progresses to a neutral and subsequently acidic state (39), however, prolonged inflammation usually leads to a decrease in the pH of the wound bed (43). This pathological pH shift not only fosters microbial growth but also impairs oxygen release from hemoglobin. There are generally two strategies for preparing pH-responsive hydrogels: one involves using copolymers with ionizable or protonatable groups, while the other utilizes chemical bonds that can be cleaved by acid or base as crosslinking units, such as Schiff base linkages (81).

**Figure 4:**
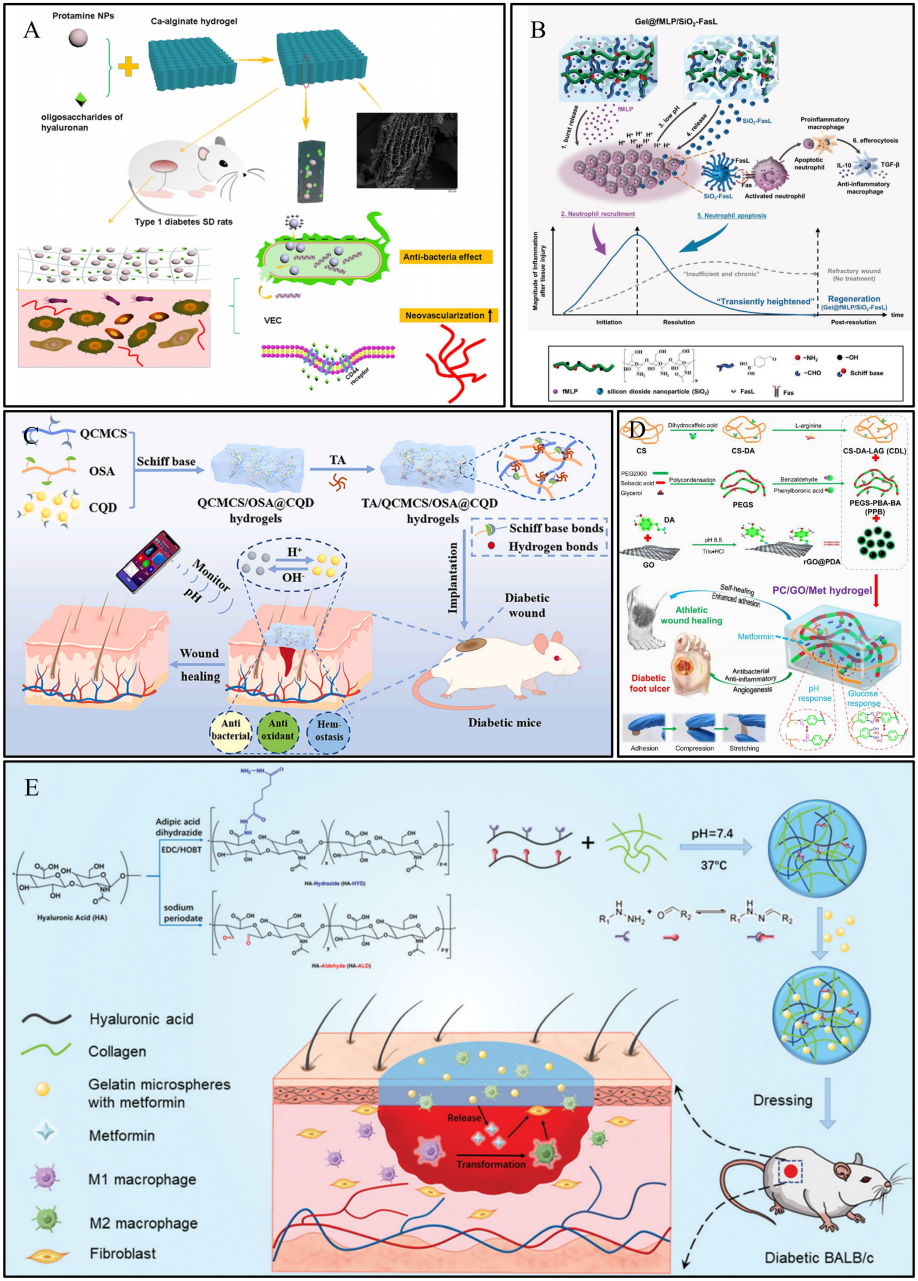
Construction process and structure of representative pH-responsive hydrogels. **(A)** Preparation of calcium alginate hydrogels and showing complete and rapid release from DWs. Reproduced from ref. (100) Copyright 2021 Springer. **(B)** Schematic illustration of the Gel@fMLP/SiO_2_-FasL for transiently heightened inflammatory response manipulation to initiate refractory wound healing. Reproduced from ref. (84) Copyright 2022 Wiley. **(C)** Schematic diagram of preparation process of TA/QCMCS/OSA@CQD hydrogels and their application for promoting diabetic wound healing and real time monitoring. Reproduced from ref. (86) Copyright 2024 Elsevier. **(D)** Schematic diagram of preparation and application of PC/GO/Met hydrogel. Reproduced from ref. (43) Copyright 2024 ACS. **(E)** Schematic illustration of the HA–COL hydrogel and its application as dressing on a full-thickness wound model in diabetic mice Reproduced from ref. (87) Copyright 2022 RSC.

Polymers such as alginate, acrylic acid, and carboxymethyl cellulose are rich in carboxylic acid groups. Calcium alginate is derived from seaweed and is highly absorbent, making it particularly suitable for treating moderately to heavily exuding wounds (82). Hydrogels prepared based on these molecules typically exhibit a higher swelling ratio under alkaline conditions, as the carboxyl groups are more readily ionized, leading to increased electrostatic repulsion (83). Wang et al. (83) developed a pH-responsive hydrogel based on alginate-calcium, loaded with protamine nanoparticles and hyaluronic acid oligosaccharides (HAO). In a medium with a pH of 3.0, the carboxyl groups in the hydrogel exist predominantly in the -COOH form, leading to chemical crosslinking between the polymers and causing the hydrogel to shrink. Conversely, in a medium with a pH of 8.0, the carboxyl groups exist as -COO−. The electrostatic repulsion between these groups causes the hydrogel to swell, a process further intensified by the formation of hydrogen bonds between -COO − and water. In an alkaline medium, the hydrogel could release 98.7% of the HAO within 8 h, which was 2.2 times the amount released in an acidic medium. Therefore, this hydrogel can specifically and effectively deliver drugs to DWs, promoting their healing (Figure 4A). Building on these principles, the teams of Liu et al. (84) and Zeng et al. (85) also constructed pH-responsive hydrogels for the on-demand release of active substances, demonstrating excellent results in promoting the repair of diabetic wound. For instance, Liu et al. prepared a hybrid biomaterial (Gel@fMLP/SiO2-FasL) by loading silica nanoparticles (SiO_2_-FasL), which combined N-formyl-methionyl-leucyl-phenylalanine (fMLP) with the recombinant protein (FasL), into a pH-responsive hydrogel. This design enabled a burst release of fMLP to rapidly recruit neutrophils, accelerating the initiation of the inflammatory response. This, in turn, led to a decrease in pH, causing the hydrogel to cleave and expose the SiO_2_-FasL. The exposed FasL then induced apoptosis in the activated neutrophils via FasL–Fas signaling. The apoptotic neutrophils were subsequently cleared by macrophages, promoting their transition to an anti-inflammatory phenotype and driving regeneration (Figure 4B).

Furthermore, leveraging the pH variations in the wound microenvironment to construct smart responsive hydrogels is also an effective strategy. For instance, the team led by Yang et al. (86) designed a pH-responsive hydrogel based on tannic acid (TA), quaternized carboxymethyl chitosan (QCMCS), and sodium alginate oxide (OSA), incorporating carbon quantum dots (CQDs) for real-time monitoring. When the skin is damaged, the wound pH rises to 7–8. This alkaline microenvironment promotes the responsive release of TA from the hydrogel; under pH 8 conditions, the cumulative release of TA reached 42.6% within 72 h, compared to only 13.2% in an acidic environment of pH 5. The released TA can exert its antioxidant and antibacterial effects to promote wound healing. Concurrently, the immobilized CQDs enable real-time monitoring of the wound’s pH recovery process through fluorescence changes, thereby allowing for an assessment of the healing status. *In vivo* results demonstrated that the wound healing rate in the hydrogel-treated group reached as high as 97% by day 12, accompanied by significant collagen deposition (73.2%) and angiogenesis levels (relative expression approximately 5 times that of the control group; Figure 4C).

In addition, many dynamic chemical bonds that can dissociate in response to acid (such as boronate ester bonds) are also common strategies for constructing pH-responsive hydrogels. Liang et al. (43) utilized Schiff base linkages between chitosan and benzaldehyde-modified polyethylene glycol-polyglycerol sebacate (Figure 4D), and Jia et al. (87) constructed dynamically crosslinked, pH-responsive hydrogels based on acylhydrazone bonds (Figure 4E). These hydrogels can release active substances on demand, providing new approaches for the design of pH-responsive hydrogel dressings for the repair of DWs.

For multifunctional hydrogels, drug release has a great impact on the wound treatment effect. With the help of changes in the pH value of the wound environment, the release of drugs can be precisely realized on demand, avoiding the frequent administration of drugs or the formation of drug resistance in the clinic. However, this also poses new challenges for the production of hydrogels. On the one hand, the pH of the hydrogel itself should be appropriate to avoid causing new irritation to the wound. On the other hand, it should be appropriately sensitive to the pH response of the wound site to ensure proper drug release.

### 5.3 Temperature-responsive hydrogels

Temperature, as a mild stimulus, is strongly associated with wound healing, as many enzyme reaction rates are temperature dependent, and temperature is also a classic indicator used to assess the clinical signs and symptoms checklist for chronic wounds, and may also serve as a trigger for responsive wound dressings (88–90). Temperature-responsive hydrogels prepared from thermo-responsive polymers, such as methylcellulose derivatives, poly (N-isopropylacrylamide; PNIPAM), poly (2-oxazoline), or poly (oxamine), can undergo rapid phase or volume changes at their upper critical solution temperature (UCST) or lower critical solution temperature (LCST) (91). Chen et al. (92) developed a temperature-responsive hydrogel patch based on N-acryloyl glycinamide, 1-vinyl-1,2,4-triazole, and PNIPAM, which was loaded with VEGF. Due to the presence of PNIPAM, the hydrogel patch could release VEGF under the stimulation of body temperature. This release reduced the expression of inflammatory factors, promoted collagen deposition and angiogenesis, and significantly accelerated wound healing in diabetic rats. Furthermore, owing to its inverse opal structure, the hydrogel patch exhibited color-sensing properties in response to temperature changes. This feature can be applied to monitor wound infections, enabling effective wound management and guiding clinical treatment.

**Figure 5:**
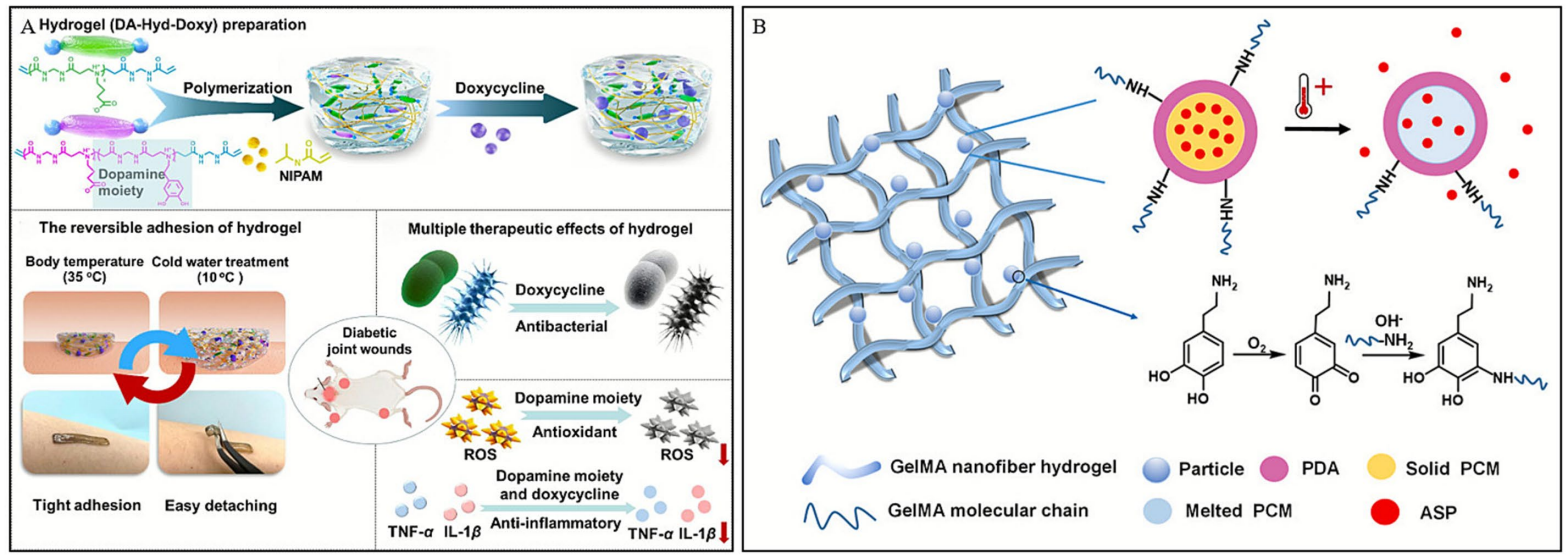
Construction process and structure of representative temperature-responsive hydrogels. **(A)** Illustration of the preparation of a hydrogel dressing (DA-Hyd-Doxy) and its mechanism for the treatment of DWs. Reproduced from ref. (93) Copyright 2025 ACS. **(B)** Schematic displays the temperature-triggered release of ASP from PDA nanoparticles. Reproduced from ref. (96) Copyright 2021 Elsevier.

In addition, some hydrogels capable of undergoing phase transitions within different temperature ranges to conform to variously shaped wounds have garnered widespread attention. Song et al. (93) developed a multifunctional hydrogel (DA-Hyd-Doxy) for the treatment of DWs, prepared by copolymerizing N-isopropylacrylamide (NIPAM) with a dopamine-containing zwitterionic poly (urethane; PAA) and encapsulating doxycycline. Utilizing the thermo-responsiveness of NIPAM, the hydrogel exhibited strong adhesion at body temperature (35 °C), whereas its adhesion could be reduced to 0 N after soaking in 10 °C water for 10 min, enabling painless removal (Figure 5A). When applied to wounds, this adhesive hydrogel, featuring painless detachment and multiple biological functions, could potentially address the limitations of existing skin adhesives that often have aggressive adhesion, and accelerate diabetic wound healing by modulating the immune microenvironment.

Besides thermo-responsive polymers, natural fatty acids also play a role in thermoresponsive drug release (94). Lauric acid (LA) and stearic acid (SA) are especially attractive owing to their biocompatibility, biodegradability and low cost among the organic natural fatty acids (95). Based on this, Zhang et al. (96) designed a temperature-responsive hydrogel dressing, GelMA-PDAASP (Figure 5B), which can achieve controlled release of drugs and is significantly better than traditional dressings, commercially available 3 M or gelatin dressings in promoting wound healing in mice.

Temperature-responsive hydrogel dressing can not only respond to the temperature of human skin, but also be regulated by the temperature of the external environment. Although the mechanism appears straightforward, stringent temperature controls are required during production, transportation, and storage. Therefore, it is necessary to fully consider the differences in temperature caused by environmental factors (e.g., region or season), as well as physiological factors (e.g., age).

### 5.4 Electro-responsive hydrogels

**Figure 6:**
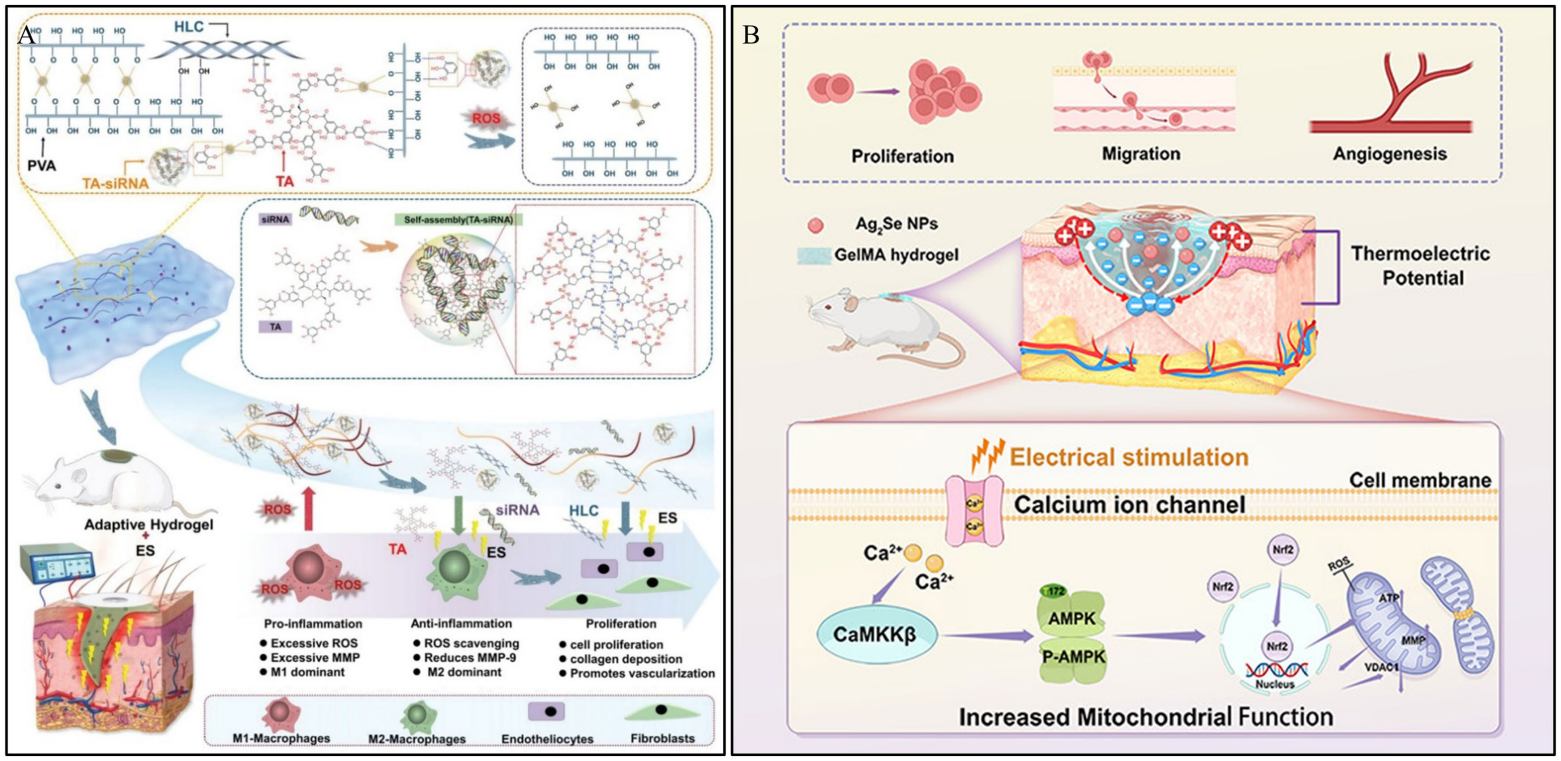
Construction process and structure of representative electro-responsive hydrogels. **(A)** The repair of DWs using the combination of ES therapy and adaptive, conductive PHTB(TA-siRNA) hydrogels. Reproduced from ref. (98) Copyright 2022 Wiley. **(B)** Schematic diagram of the synthesis and biological mechanism of the Ag_2_Se@GelMA hydrogel-based ES device for DWs. Reproduced from ref. (99) Copyright 2025 ACS.

Electrical conductivity is an intrinsic property of the skin, and it can accelerate various stages of the chronic wound healing process. It promotes wound healing by guiding keratinocyte migration, enhancing epithelial regeneration, directing dermal angiogenesis, and modulating the expression of multiple factors associated with wound healing (97). In this context, Lei et al. (98) first prepared TA-siRNA nanogels based on tannic acid (TA) and small interfering RNA (siRNA). Subsequently, the TA-siRNA nanogels were crosslinked with polyvinyl alcohol (PVA), human-like collagen (HLC), TA, and borax via boronate ester bonds to fabricate an adaptable, conductive PHTB hydrogel with ROS-scavenging capabilities. The results demonstrated that electrical stimulation (ES) could promote the release of TA-siRNA nanogels from the PHTB hydrogel (Figure 6A). This release silenced the matrix metalloproteinase 9 gene (MMP-9), which is highly expressed in the pro-inflammatory microenvironment of chronic wounds. Consequently, this reduced the levels of MMP-9 protein, promoted macrophage polarization, collagen production, and angiogenesis, thereby accelerating diabetic wound healing.

Thermoelectric technology, which can generate electricity by harnessing the temperature difference between the skin and the surrounding environment without external energy input, offers a viable pathway for ES therapy. Qin et al. (99) developed a thermoelectric hydrogel, Ag_2_Se@g-methacrylated gelatin (Ag_2_Se@GelMA), possessing high room-temperature thermoelectric properties, to be used as self-powered ES for wound repair. This thermoelectric hydrogel can accelerate wound closure by amplifying endogenous electric fields, thereby promoting cell proliferation, migration, and angiogenesis. *In vitro* experiments demonstrated that the ES generated by the hydrogel activates voltage-gated calcium ion channels, increasing intracellular Ca^2+^ levels and enhancing mitochondrial function via the Ca^2+^/CaMKKβ/AMPK/Nrf2 pathway. This cascade reaction improves mitochondrial dynamics and angiogenesis, thus accelerating tissue regeneration (Figure 6B). These combined effects expedite wound healing and support effective skin tissue reconstruction, offering insights for the repair of DWs.

Electro-responsive hydrogels offer a unique therapeutic advantage by restoring the endogenous electric fields disrupted in diabetic wounds and enabling precise, on-demand drug release to regulate cell migration and mitochondrial function. However, their clinical translation is currently limited by the potential cytotoxicity of conductive nanomaterials (e.g., Ag₂Se) and the technical challenge of maintaining stable conductivity within a swelling, wet wound microenvironment. Furthermore, while self-powered thermoelectric strategies reduce dependence on cumbersome external power sources, their efficiency is often constrained by environmental temperature fluctuations, necessitating future research into more stable, biodegradable, and flexible bio-electronic interfaces to ensure patient safety and compliance.

### 5.5 ROS-responsive hydrogels

**Figure 7:**
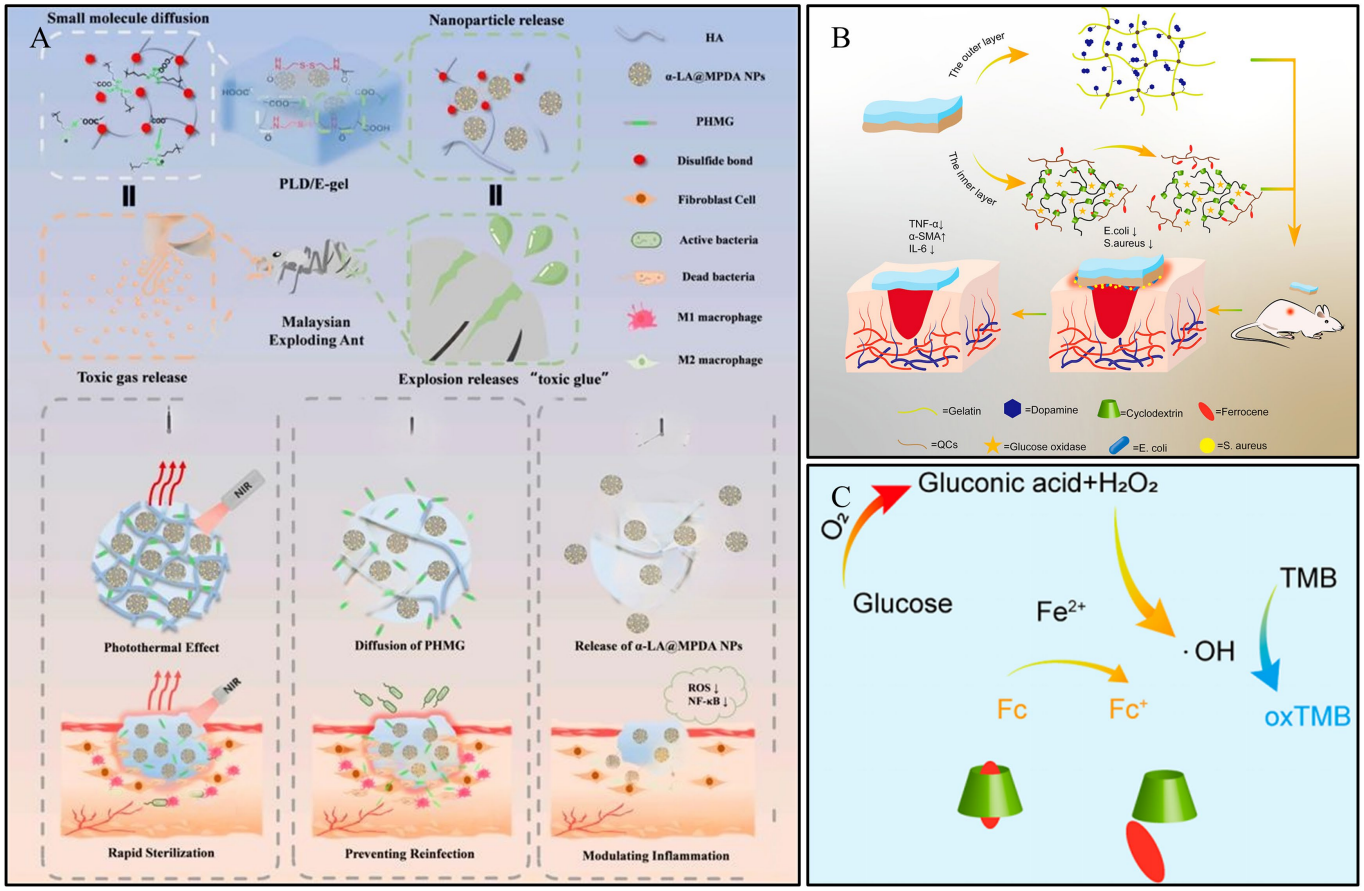
Construction process and structure of representative ROS-responsive hydrogels. **(A)** Fabrication of PLD/E-gel and its application on diabetic SD rat with full-thickness skin defect and infections. Reproduced from ref. (101) Copyright 2024 Elsevier. **(B)** Construction of double-layer hydrogel for DWs. Reproduced from ref. (102) Copyright 2023 ACS. **(C)** Diagram of the cascade reaction of a bilayer hydrogel. Reproduced from ref. (102) Copyright 2023 ACS.

ROS are a class of highly reactive ions produced in the human body. They play a crucial role in regulating cellular signaling pathways, inflammation, and cell proliferation (90). However, in the chronic DWs environment, the redox balance is disrupted, leading to oxidative stress, which inhibits wound healing (100). ROS-responsive hydrogels are designed to cleave and accelerate drug release when ROS levels are elevated, thereby promoting wound healing. Sun et al. (101) developed an ROS-responsive “explosive hydrogel” (PLD/E-gel) with a size-dependent sequential release effect, based on the water-soluble small molecule polyhexamethylene guanidine (PHMG, P) and mesoporous polydopamine nanoparticles loaded with alpha-lipoic acid (*α*-LA@MPDA NPs, LD). During the water absorption and swelling of the PLD/E-gel, PHMG effectively prevents wound reinfection through water-soluble diffusion. Subsequently, as the PLD/E-gel degrades, the sub-micron-sized α-LA@MPDA NPs are released from the crosslinked network, scavenging excessive ROS and modulating the inflammatory cascade. The PLD/E-gel accelerates the healing of infected DWs in SD rats by rapidly killing bacteria, inhibiting bacterial recolonization, alleviating wound inflammation, and promoting collagen deposition (Figure 7A). Tan et al. (102) developed a novel bilayer hydrogel designed to regulate ROS levels, offering specific benefits for diabetic wound healing. The inner layer contains GOx, ferrocene-modified quaternary ammonium CS, and poly (*β*-cyclodextrin), which generates hydroxyl radicals (-OH) for antimicrobial action during wound recovery (Figure 7B). The layer was designed to break down gradually. The outer layer, made of gelatin and dopamine, helps to eliminate ROS in the later stages of wound repair. The ability of the hydrogel to modulate ROS in a two-stage approach to programmed diabetic wound healing was validated by antimicrobial, ROS removal, and wound healing tests (Figure 7C). This hydrogel dressing shows exceptional potential for advanced treatment of diabetic wounds.

ROS-responsive hydrogels offer a targeted therapeutic strategy by scavenging the excessive reactive oxygen species that drive chronic inflammation and triggering the on-demand release of therapeutic agents. However, a critical limitation lies in the delicate balance of ROS homeostasis; since physiological levels of ROS are essential for cell signaling and bacterial defense, indiscriminate scavenging may inadvertently hinder normal healing processes. Additionally, the clinical translation of these systems is challenged by the long-term biosafety concerns of functional nanomaterials (e.g., MPDA nanoparticles) and the inherent instability of oxidation-sensitive chemical linkers during storage and transport.

### 5.6 Enzyme-responsive hydrogels

**Figure 8:**
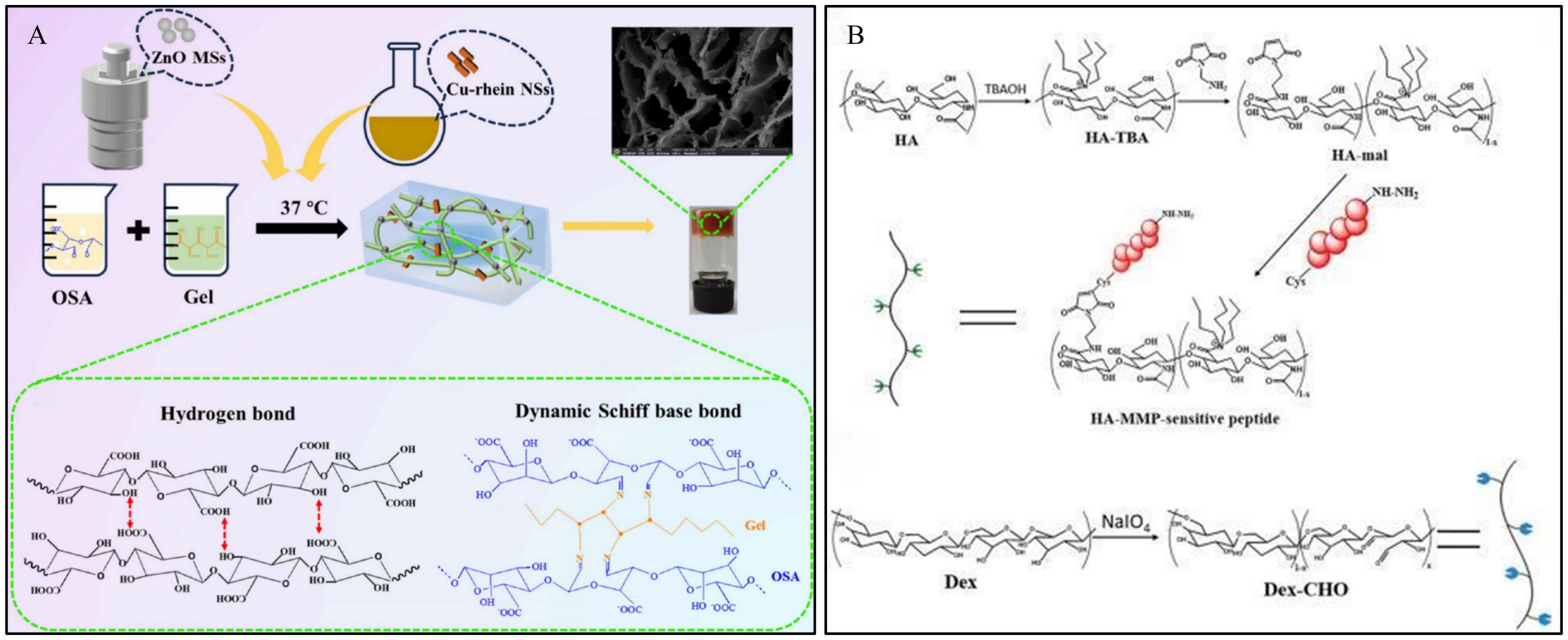
Construction process and structure of representative enzyme-responsive hydrogels. **(A)** Outline of the preparation process of Cu-Rhein NSs, ZnO MSs, and smart hydrogels. Reproduced from ref. (106) Copyright 2024 ACS. **(B)** Schematic illustration of the construction of MMP-cleavable hydrogel. Reproduced from ref. (62) Copyright 2022 Elsevier.

Enzyme-responsive systems offer higher efficiency and specificity compared to other responsive systems (103). With the development of nanotechnology and catalytic science, nanozymes have attracted widespread attention due to their advantages such as functional diversity, high stability, tunable catalytic activity, and ease of large-scale production (104). Zn^2+^ has been shown to influence the activity of MMP-9 positively (105). Feng et al. (106) designed a hydrogel with intrinsic ultra-long pores, smart controlled release, and dynamic adhesion properties for diabetic wound healing, based on rhein-based bionic nanozymes (Cu-rhein NSs) and zinc oxide microspheres (ZnO MSs). The Cu-rhein NSs and ZnO MSs were synthesized onto an ECM-mimicking dual-network smart hydrogel, constructed from sodium silicate, gelatin, and borax (Figure 8A). This hydrogel possesses synergistic antibacterial properties, clearing bacteria from the wound while forming a protective barrier against external bacteria and pathogens. Furthermore, it can intelligently release the bionic nanozymes to effectively eliminate bacteria, reduce inflammatory responses, scavenge multiple free radicals, and accelerate the healing of infected DWs.

Diabetic wounds typically exhibit MMP overexpression compared to normal wounds, thereby delaying the healing process. Clinical wound healing can be accelerated by MMP-responsive hydrogel dressings, which can cause MMP to become inactive or protect cells by competing for substrates. In their study, Li et al. (62) developed an HA-based hydrogel for diabetic wound healing that was MMP responsive and loaded with DFO, a drug that is beneficial for wound repair but is limited by its toxicity and short half-life. By modifying HA with maleimide and linking the MMP-cleavable peptide, they designed an enzyme-responsive hydrogel that was crosslinked with varying amounts of ODex to form the final gel (Figure 8B). Application of this hydrogel to wounds in diabetic rats significantly improved the rate of wound closure, the process of new skin formation (epithelialization), and the development of new blood vessels (angiogenesis) compared to a control hydrogel containing a non-cleavable peptide. This finding emphasizes the efficacy of MMP-responsive hydrogels in delivering effective therapeutic substances, such as DFO, directly to diabetic wounds, thereby avoiding the problems associated with their toxicity and instability.

Hydrogel dressings that respond to specific enzymes can address the problem of MMP overexpression in diabetic wounds. These dressings can be designed to be removed as wound healing progresses, thereby improving safety. Nonetheless, factors such as the microenvironment of the wound and the pH of the dressing remain important considerations, as they can affect enzyme activity. These aspects must be carefully considered during the design, manufacture and clinical application of such dressings (107).

### 5.7 Multi-responsive hydrogels

Hydrogel dressings with a single response mechanism often struggle to achieve the desired high efficiency and precise targeted drug delivery, resulting in suboptimal therapeutic effects on chronic wounds (108). By constructing dual/multi-stimuli-responsive hydrogel wound dressings, the release of active substances such as drugs can be more precisely controlled, better meeting the needs of different stages of wound healing.

In chronic DWs, persistent high glucose levels and the inflammatory environment impede the normal wound healing process. Dai et al. (109) engineered a sophisticated multi-responsive platform by constructing a dynamic network crosslinked via Schiff base and boronate ester bonds. Leveraging the pH-sensitivity of these dynamic linkages (mechanisms detailed in Section 3.2), the hydrogel undergoes programmed degradation upon exposure to the acidic diabetic wound microenvironment. This distinct dual-dynamic strategy not only ensures structural adaptability but also synergistically facilitates the release of bioactive agents, thereby modulating macrophage polarization. The study demonstrated that the hydrogel enhanced angiogenesis by upregulating the expression levels of angiogenesis-related factors, such as hypoxia-inducible factor-1*α* (HIF-1α), vascular endothelial growth factor (VEGF), CD31, and α-SMA. This significantly accelerated wound healing in streptozotocin (STZ)-induced diabetic mice. This multi-responsive, multifunctional hydrogel provides a locally specific, dual-responsive drug release strategy for diabetic foot ulcers.

PBA-based hydrogels, possessing good stability and no immune rejection in the human body, have long been explored for glucose-responsive insulin delivery. Guo et al. (110) synthesized a multi-stimuli-responsive conductive hydrogel by copolymerizing the zwitterionic monomer sulfobetaine, the thermosensitive monomer N-isopropylacrylamide, and the glucose-responsive monomer methacrylamide phenylboronic acid. Based on this hydrogel, they developed an ionic skin sensing system capable of continuous, real-time monitoring of three indicators: temperature, strain, and glucose.

**Figure 9:**
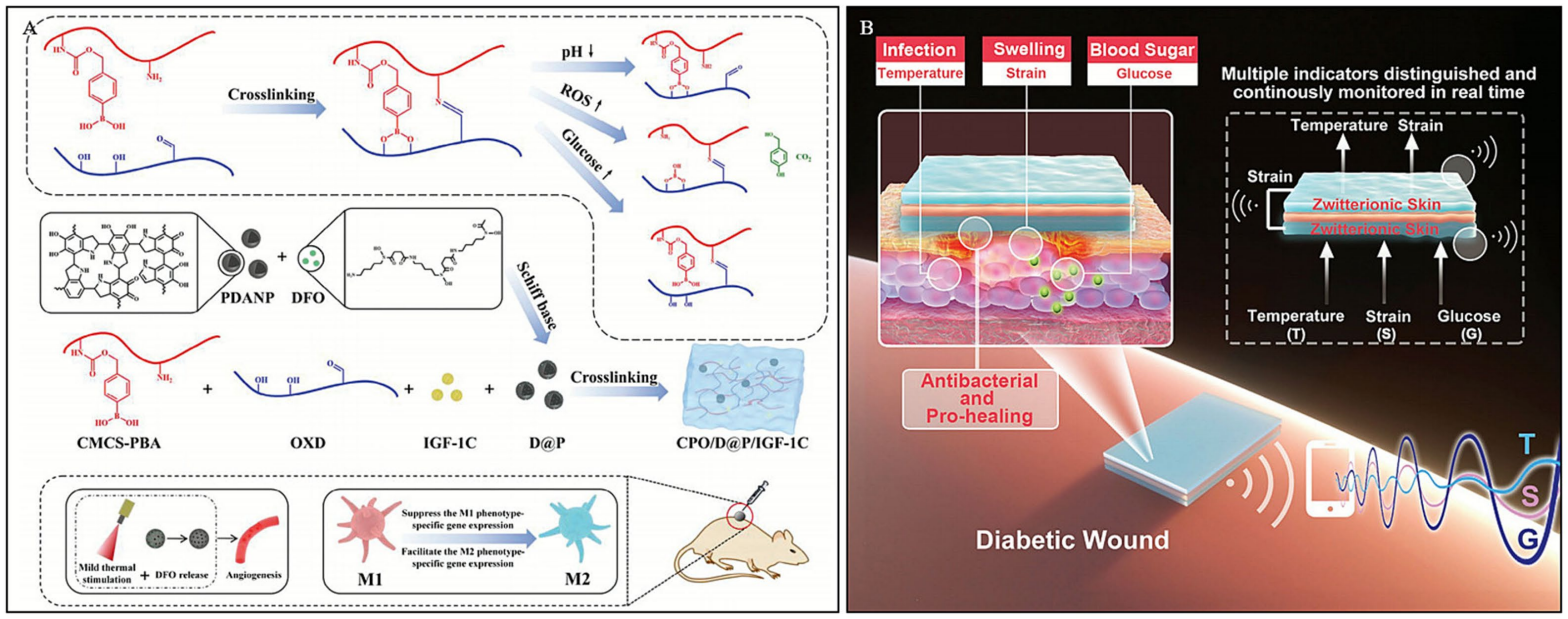
Construction process and structure of representative multi -responsive hydrogels. **(A)** Synthesis diagram of CPO/D@P/IGF-1C hydrogel and mechanism of DWs promoted by the hydrogel combined with mild heat stimulation. Reproduced from ref. (109) Copyright 2024 Wiley. **(B)** Scheme illustration of the sandwich-structured sensor based on multi-response zwitterionic skin for multiple sensation and pro-healing of DWs. Reproduced from ref. (110) Copyright 2021 Wiley.

Multi-stimuli-responsive hydrogels offer a distinct therapeutic advantage by mimicking the dynamic complexity of diabetic wounds, enabling synergistic effects—such as simultaneous inflammation modulation and angiogenesis—that single-response systems often fail to achieve. However, their clinical translation is significantly impeded by the intricate synthesis processes required, which pose substantial challenges for reproducibility, large-scale manufacturing, and cost-effectiveness. Moreover, the regulatory pathway for such complex multicomponent systems is arduous; ensuring the long-term stability and biosafety of multiple interacting functional modules remains a critical barrier to their approval and commercial adoption (111, 112) (Figure 9).

## 6 Challenges and future perspectives

DWs represent a complex clinical challenge characterized by a dynamic and hostile microenvironment. As summarized in this review, stimuli-responsive hydrogels offer a distinct advantage over traditional passive dressings by enabling the “on-demand” release of therapeutic agents in response to specific pathological cues, such as pH, glucose, ROS, and enzymes. These smart biomaterials not only improve treatment precision but also actively modulate the wound environment. However, despite the rapid development of these functional hydrogels, several fundamental hurdles currently hinder their translation from bench to bedside.

Firstly, there is a significant discrepancy between preclinical animal models and clinical reality. Most efficacy data are currently derived from rodent models, which possess loose skin and heal primarily by contraction. This differs fundamentally from human skin, which is tight and heals via re-epithelialization. Consequently, the predictive value of these models for clinical outcomes in DWs patients is often limited. To address this, future research should prioritize advanced preclinical models, such as porcine models or ex vivo human skin models. Since porcine skin shares greater anatomical and physiological similarities with human skin (113), these models would provide more reliable validation of hydrogel efficacy.

Secondly, despite the promising preclinical results summarized in this review, the translation of stimuli-responsive hydrogels to clinical practice remains in its infancy. While traditional hydrogels are widely used in wound care, most “smart” hydrogels are still restricted to the laboratory phase, with very few entering clinical trials. Many responsive systems rely on complex nanomaterials (e.g., silver nanoparticles, quantum dots) or enzymes (e.g., glucose oxidase), for which long-term cytotoxicity and organ accumulation remain unresolved concerns (114). Therefore, future designs must balance functional complexity with manufacturing scalability. While multi-responsiveness is scientifically intriguing, simpler designs with robust mechanisms are more likely to achieve regulatory approval and commercial viability (112).

Thirdly, the integration of emerging technologies offers a promising path forward. The synthesis of multi-stimuli-responsive hydrogels often involves intricate, multi-step chemical reactions, making large-scale production difficult and costly compared to traditional dressings (115). Environmental factors during production, storage, and transportation—such as temperature fluctuations and oxidizing substances—can further compromise gel formation and drug release behavior. In addition, standard sterilization methods (e.g., gamma irradiation, ethylene oxide, or high-pressure steam) can easily denature protein-based components (like GOx or Con A) or disrupt the precise crosslinking networks required for responsiveness (116). Therefore, developing non-destructive sterilization protocols or self-sterilizing materials is essential for clinical adoption.

Finally, the integration of emerging technologies offers a promising path forward. To accelerate material development, AI-driven design utilizing machine learning algorithms can be employed to predict optimal polymer ratios and screen formulations tailored to the specific DWs microenvironment. Moreover, the next generation of hydrogels should evolve toward “theranostics”—integrating diagnosis and treatment. By incorporating visual or electronic feedback systems (e.g., colorimetric sensors), these smart dressings could inform clinicians of wound status in real-time. We anticipate that overcoming these barriers will pave the way for personalized, intelligent wound care solutions that significantly reduce the burden of amputation in diabetic patients.
