# Supplementary material for: Application and development of stimulus-responsive hydrogel biomaterials for diabetic wound healing: a literature review
Source: Front Med (Lausanne). 2026 Jan 8;12:1740559. doi: 10.3389/fmed.2025.1740559 (PMC12823876; doi:10.3389/fmed.2025.1740559)
Supplement: Supplementary file 1 [file Table_1.DOCX]

| Stimulus | Key Material Composition | Therapeutic Payload | Detailed mechanism | Reference |
| --- | --- | --- | --- | --- |
| Glucose | Concanavalin A (Con A) | insulin | Free glucose can competitively bind with Con A within the hydrogel, leading to the dissociation of the hydrogel system, | Yin et al. (2019) |
|  | Glucose oxidase (GOx) | Antimicrobial peptides (AMP), Vascular endothelial growth factor (VEGF) | In a high-glucose wound environment, GOX catalyzes glucose to produce gluconic acid, which triggers the hydrolysis/breakage of the Schiff base bonds between ODex and QCS, causing the rapid degradation of the hydrogel network and the subsequent release of loaded drugs (AMP and VEGF). | Wang et al. (2025) |
|  | Phenylboronic acid-modified gelatin (AP), Sodium alginate (SA) | insulin | In a high-glucose environment, glucose molecules competitively bind to the PBA groups, disrupting the original ester bonds. This leads to the dissociation/dissolution of the hydrogel network | Zhou et al. (2026) |
| pH | Alginate calcium | Protamine, Hyaluronan oligosaccharides, (HAO) | In the alkaline conditions (pH 8.0), carboxylic groups in the hydrogel ionize to -COO^-^, causing electrostatic repulsion. This leads to significant swelling and loosening of the hydrogel network, triggering the rapid release of the loaded drugs | Wang et al. (2019) |
|  | Chitosan, 4-Formylphenylboronic acid (FPBA) | fMLP, Fas Ligand (FasL) | The burst release of fMLP recruits neutrophils, generating an acidic environment that triggers the hydrolysis of borate ester bonds, leading to hydrogel dissociation and the release of SiO_2_-FasL. | Liu et al. (2022) |
|  | Incorporating carbon quantum dots (CQDs) | Tannic acid (TA), CQDs | CQDs exhibit pH-dependent fluorescence/color changes (linear correlation with RGB signals), visually indicating wound status (infection/alkaline vs. healing). | Yang et al. (2024) |
|  | Phenylboronic acid & benzaldehyde functionalized polymer (PEGS-PBA-BA) | Metformin (Met), Graphene oxide (GO) | Acidic pH (e.g., pH 5.5) triggers the hydrolysis/dissociation of Schiff base bonds (formed between benzaldehyde and amino groups) | Liang et al. (2022) |
|  | Oxidized Hyaluronic Acid (HA-ALD), Hydrazide Hyaluronic Acid (HA-HYD) | Met, Collagen (COL) | The hydrogel network is crosslinked by dynamic hydrazone bonds (formed between aldehyde and hydrazide groups). Acidic wound pH (~6.5) triggers their hydrolysis, leading to hydrogel degradation and the release of Metformin and Collagen. | Jia et al. (2022) |
| Temperature | N-isopropylacrylamide (NIPAM) | VEGF；Doxycycline | Inflammation-induced temperature rise (37℃) triggers PNIPAM phase transition and shrinkage, mechanically squeezing out encapsulated VEGF. | Chen et al. (2022) ；Song et al. (2025) |
|  | Lauric acid (LA), stearic acid (SA) | Aspirin (ASP) | The LA/SA eutectic mixture melts above 39℃ (simulating inflammation), triggering rapid, "on-off" Aspirin release from the previously solid matrix | Zhang et al. (2021) |
| Electric | Polyvinyl alcohol (PVA),  Human-like collagen (HLC), TA, Borax | siRNA | The external electric field stimulates the movement of the charged TA-siRNA nanogels, accelerating their release from the hydrogel network. It also promotes blood circulation/tissue fluid flow, which accelerates hydrogel degradation and further payload release. | Lei et al. (2022) |
|  | Ag₂Se, Gelatin Methacryloy (GelMA) | Electrical stimulation (ES) (Generated in situ by the hydrogel itself) | The hydrogel utilizes the natural temperature difference between the warm wound surface and the cooler ambient environment (maintained by hydrogel evaporation). This ΔT drives the directional movement of electrons in the Ag₂Se semiconductor component, generating a thermoelectric potential/ES without external power. This ES activates voltage-gated calcium channels to promote healing. | Qin et al. (2025) |
| ROS | Sulfhydryl-modified hyaluronic acid (HA-SH) | Polyhexamethylene guanidine，α-lipoic acid， | In the presence of high levels of ROS in diabetic wounds), these disulfide bonds are oxidized and cleaved. This cleavage disrupts the hydrogel network structure, leading to its degradation and the sequential release of the loaded payloads | Sun et al. (2024) |
|  | Ferrocene, β-Cyclodextrin (β-CD) | 1. Glucose Oxidase (Gox)  2. Hydroxyl Radicals (·OH)  3. α-LA@MPDA NPs | 1. ROS Generation: Gox converts glucose to H_2_O_2_  2. Fenton Reaction: The Ferrocene (Fc) group (Fe^2+^) catalyzes H_2_O_2_ into highly antibacterial hydroxyl radicals (·OH).  3. Network Collapse: During this process, Fc (Fe^2+^) is oxidized to Fc^+^ (Fe^3+^). The oxidized Fc^+^ cannot form a stable inclusion complex with β-CD. This disrupts the host-guest crosslinking, causing the inner hydrogel network to dissociate/collapse, releasing the loaded α-LA@MPDA NPs. | Tan et al., (2023) |
| Enzyme | Oxidized Sodium Alginate (OSA), Borax, Gelatin. | biomimetic nanozyme based on rhein (Cu-rhein NSs), zinc oxide microspheres (ZnO MSs) | The hydrogel network is crosslinked by dynamic Schiff base bonds (between OSA aldehyde and gelatin amino groups) and borate ester bonds. In the acidic environment of chronic wounds, these bonds (especially Schiff base) are unstable and break. This leads to the dissociation of the hydrogel structure and release of the loaded Cu-rhein nanozymes and ZnO MSs. | Feng et al. (2024) |
|  | Hyaluronic acid grafted with the MMP-sensitive peptide sequence: CPLGLAG-NH-NH2 | Deferoxamine (DFO) | The hydrogel network is crosslinked via the MMP-cleavable peptide sequence (**CPLGLAG**). In the presence of *Matrix* Metalloproteinase-2 (MMP-2), which is highly elevated in diabetic wounds, this specific peptide sequence is **enzymatically cleaved**. This cleavage disrupts the hydrogel network, leading to its degradation and the subsequent release of the loaded DFO drug on demand. |  |
| Multi | Oxidized dextran (OXD), Phenylboronic acid-modified carboxymethyl chitosan (CMCS-PBA), Polydopamine nanoparticles (PDANP) | Deferoxamine (DFO), Insulin-like growth factor 1 C domain (IGF-1C) | 1. pH Response: The Schiff base (imine) bonds crosslinking the hydrogel are unstable and cleave in the acidic microenvironment (pH 6.5) of diabetic wounds, triggering drug release.  2. Glucose Response: High glucose levels competitively bind with phenylboronic acid groups, dissociating the phenylboronate ester bonds and loosening the hydrogel network.  3. ROS Response: High levels of ROS oxidize the phenylboronate ester bonds, causing network degradation and payload release.  4. Photothermal Response: PDANP converts NIR laser energy into mild heat (40−41℃), which promotes macrophage polarization and angiogenesis. | Dai et al. (2024) |
|  | NIPAM, 3-(acrylamido)phenylboronic acid (MPBA), Insulating elastomer (VHB) | Zwitterionic matrix (SBMA) | 1.Temperature: Detected by Resistance (R_Upper_) changes in the upper gel via NIPAM 's thermosensitivity  2. Glucose: Detected by Resistance (R_Lower_) changes in the lower gel. MPBA binds glucose, expelling water and increasing ion conductivity. | Guo et al. (2021) |

Table S1. A comprehensive list with detailed mechanisms
